# Physiological oxygen conditions enhance the angiogenic properties of extracellular vesicles from human mesenchymal stem cells

**DOI:** 10.1186/s13287-023-03439-9

**Published:** 2023-08-23

**Authors:** Jolene Phelps, David A. Hart, Alim P. Mitha, Neil A. Duncan, Arindom Sen

**Affiliations:** 1 Pharmaceutical Production Research Facility, Schulich School of Engineering, University of Calgary, 2500 University Drive N.W., Calgary, AB T2N 1N4 Canada; 2 Department of Chemical and Petroleum Engineering, Schulich School of Engineering, University of Calgary, 2500 University Drive N.W., Calgary, AB T2N 1N4 Canada; 3 Department of Biomedical Engineering, Schulich School of Engineering, University of Calgary, 2500 University Drive N.W., Calgary, AB T2N 1N4 Canada; 4 Department of Surgery, Cumming School of Medicine, University of Calgary, 3330 Hospital Drive N.W., Calgary, AB T2N 4N1 Canada; 5 Department of Clinical Neurosciences, Cumming School of Medicine, University of Calgary, 3300 Hospital Drive N.W., Calgary, AB T2N 4N1 Canada; 6 Department of Civil Engineering, Schulich School of Engineering, University of Calgary, 2500 University Drive N.W., Calgary, AB T2N 1N4 Canada; 7 Musculoskeletal Mechanobiology and Multiscale Mechanics Bioengineering Lab, Department of Civil Engineering, Schulich School of Engineering, University of Calgary, 2500 University Drive N.W., Calgary, AB T2N 1N4 Canada; 8 McCaig Institute for Bone and Joint Health, Cumming School of Medicine, University of Calgary, 3280 Hospital Drive N.W., Calgary, AB T2N 4Z6 Canada

**Keywords:** Extracellular vesicles, Angiogenesis, Adipose stem cells, Cerebral microvascular endothelial cells, Stroke, Cell-free therapy, Hypoxia, Bioprocessing, Exosomes, Microvesicles

## Abstract

**Background:**

Following an ischemic injury to the brain, the induction of angiogenesis is critical to neurological recovery. The angiogenic benefits of mesenchymal stem cells (MSCs) have been attributed at least in part to the actions of extracellular vesicles (EVs) that they secrete. EVs are membrane-bound vesicles that contain various angiogenic biomolecules capable of eliciting therapeutic responses and are of relevance in cerebral applications due to their ability to cross the blood–brain barrier (BBB). Though MSCs are commonly cultured under oxygen levels present in injected air, when MSCs are cultured under physiologically relevant oxygen conditions (2–9% O_2_), they have been found to secrete higher amounts of survival and angiogenic factors. There is a need to determine the effects of MSC-EVs in models of cerebral angiogenesis and whether those from MSCs cultured under physiological oxygen provide greater functional effects.

**Methods:**

Human adipose-derived MSCs were grown in clinically relevant serum-free medium and exposed to either headspace oxygen concentrations of 18.4% O_2_ (normoxic) or 3% O_2_ (physioxic). EVs were isolated from MSC cultures by differential ultracentrifugation and characterized by their size, concentration of EV specific markers, and their angiogenic protein content. Their functional angiogenic effects were evaluated in vitro by their induction of cerebral microvascular endothelial cell (CMEC) proliferation, tube formation, and angiogenic and tight junction gene expressions.

**Results:**

Compared to normoxic conditions, culturing MSCs under physioxic conditions increased their expression of angiogenic genes *SDF1* and *VEGF,* and subsequently elevated VEGF-A content in the EV fraction. MSC-EVs demonstrated an ability to induce CMEC angiogenesis by promoting tube formation, with the EV fraction from physioxic cultures having the greatest effect. The physioxic EV fraction further upregulated the expression of CMEC angiogenic genes *FGF2*, *HIF1*, *VEGF* and *TGFB1*, as well as genes (*OCLN* and *TJP1*) involved in BBB maintenance.

**Conclusions:**

EVs from physioxic MSC cultures hold promise in the generation of a cell-free therapy to induce angiogenesis. Their positive angiogenic effect on cerebral microvascular endothelial cells demonstrates that they may have utility in treating ischemic cerebral conditions, where the induction of angiogenesis is critical to improving recovery and neurological function.

**Supplementary Information:**

The online version contains supplementary material available at 10.1186/s13287-023-03439-9.

## Background

Events such as ischemic stroke and traumatic brain injury can lead to limited blood supply in certain areas of the brain, preventing an adequate supply of oxygen and nutrients to carry out essential brain functions in those regions. The resulting hypoxic conditions have been reported to be a major contributing factor in the development of neurological deficits [1]. Angiogenesis (i.e., new blood vessel formation) within the brain has been reported to promote neurogenesis and is considered critical for post-ischemic functional recovery [2]. Thus, there is significant interest in developing therapeutic approaches that upregulate cerebral angiogenesis.

Mesenchymal stem cells (MSCs) are unspecialized adult cells that are widely recognized for their therapeutic benefits in regenerative medicine, including pro-angiogenic effects following ischemic injury or in ischemic diseases [3–6]. Although MSCs can be isolated from various tissues within the body, those derived from adipose tissue have been shown to induce the greatest angiogenic effects [7, 8]. These effects can be largely attributed to the paracrine activity of MSCs, and in particular via the actions of membrane-bound nanoparticles called extracellular vesicles (EVs) that they release [9]. EVs travel to target cells where they deliver their contents, which include biomolecules such as growth factors and microRNAs (miRNAs) capable of eliciting therapeutic responses. EVs encompass exosomes which are formed and released through the inward budding of multivesicular bodies, and microvesicles which bud from the plasma membrane. Exosome-rich and microvesicle-rich populations derived from MSCs have each been shown to independently promote angiogenesis [10–12]; however, the International Society for Extracellular Vesicles (ISEV) recommends to use the term EVs to collectively describe populations of exosomes and microvesicles, as current methods to separate these two populations are not exclusively able to sort one from the other [13]. EVs, which are nonliving, as opposed to viable MSCs, offer the promise of similar functional outcomes with a higher safety profile, and have the ability to cross biological barriers such as the blood–brain barrier (BBB), making them of exceptional interest for the treatment of brain-related disorders [14–16].

The biomolecular contents of a population of EVs depends on the source from which the parent MSCs were obtained (i.e., tissue type, donor/patient characteristics and pathological state) and the conditions under which the cells were cultured (i.e., low/high oxygen, exposure to fluid shear, medium composition, etc.) [17]. In traditional culture methods, cells are exposed to normoxic conditions, which refer to a humid environment containing 5% CO_2_ in air (20.9% O_2_). Considering the partial pressures of CO_2_ and water vapor, the headspace above the culture media sits at 18.6% O_2_ at sea level, or 18.4% O_2_ at higher elevations such as in Calgary (elevation ~ 1045 m above sea level) where the present studies were performed. In vivo, cells in adipose tissue are typically exposed to oxygen concentrations of 2–8% O_2_ [18], which can be termed physioxic conditions. Thus, relative to their native environment, the normoxic conditions typically found in culture could be considered severely hyperoxic for MSCs. Culturing MSCs at lower oxygen concentrations, referred to as hypoxic conditions (i.e., 1–5% O_2_ in the headspace), has been shown to enhance their angiogenic potential [19–22], and their production of pro-angiogenic and pro-survival factors [22–24]. This effect has been attributed to the activation of hypoxia-inducible factors (HIFs) which are typically degraded at higher oxygen levels. Indeed, through the overexpression of *HIF1*, several studies have demonstrated enhanced survival and higher angiogenic capacity of MSCs [25–29]. Moreover, when exposed to MSC-derived EVs from hypoxic culture, human umbilical vein endothelial cells exhibited higher EV uptake rates, a greater magnitude of subsequent tube formation [24, 30, 31], and improved neovascularization in a mouse model of fat grafting [24] compared to MSC-EVs from normoxic cultures.

Secondly, culture medium commonly contains serum. Serum is an undefined mixture of hormones and growth factors and contains many of the nutrients essential to cell growth. However, serum also contains clotting-generated peptides that cells would normally only see during blood coagulation and may alter the normal condition of some cells [32]. Further, the use of serum for the generation of clinical products is undesirable due to its inherent variability between batches, the risk of contamination with harmful pathogens, and the risk of immune rejection if components from the serum remain in the cells or EVs prior to transplantation [33]. The use of serum for EV production is further limited by the fact that serum contains its own EVs which co-isolate with MSC-derived EVs during the isolation process. To overcome these challenges, chemically defined serum-free culture media can be used, where the defined nature reduces the variability between batches of cells (i.e., increases reproducibility—important in clinical applications), enables precise optimization and control of cell characteristics, and has been found to produce MSCs with increased proliferation rates and MSC-EVs with increased therapeutic potential [34, 35].

Cerebral microvascular endothelial cells (CMECs) are responsible for the maintenance of the BBB and play a key role in injury response such as inflammation, angiogenesis, and the release of trophic factors [36]. Understanding how CMECs individually respond to MSC-derived EVs in vitro is an important step toward determining the clinical utility of MSC-EVs. In vivo, administration of MSC-derived EVs in preclinical animal studies has been shown to reduce neurological impairment and upregulate angioneurogenesis following cerebral ischemia/stroke [37–39] and after TBI [12]. Compared to MSCs, MSC-EVs demonstrated increased behavioral improvements in a rat stroke model [40]. In vitro, MSC-EVs from normoxic cultures have demonstrated upregulated angiogenesis in CMECs [41, 42], while work by Gregorius et al*.* [43] indicated that only EVs from hypoxic cultured MSCs (1% O_2_) could enhance CMEC angiogenesis and survival.

The aim of the present study was to compare the EV fraction harvested from human adipose MSCs grown in clinically relevant serum-free normoxic (18.4% O_2_) and physioxic (3% O_2_) conditions and to evaluate their subsequent angiogenic effect on human CMECs in vitro. Understanding the best culture conditions to process EVs for the generation of a cell-free therapy, in particular for the survival and function of CMECs, is an important step toward the clinical translation of MSC-derived EVs following ischemic brain injury.

## Materials and methods

### MSC culture

Human adipose-derived MSCs were isolated enzymatically from abdominal subcutaneous adipose tissues (female, age 20–30, BMI within normal range, abdominoplasty performed by a surgeon at the Foothills Hospital in Calgary, AB, CA) and characterized as described previously [44]. MSCs were serially expanded in serum-free PPRF-msc6 [35] under normoxic (18.4% O_2_) conditions. At each passage, the cells were inoculated at 5000 cells/cm^2^ into 12 mL of PPRF-msc6 in T75 flasks. Cells were passaged every 72 h. For EV collection, the growth medium from the MSC cultures was removed 72 h post-inoculation, and cells were washed twice with Dulbecco’s phosphate-buffered solution (DPBS). 12 mL of EV collection medium (fresh PPRF-msc6 ultracentrifuged at 105,000 g for 18.5 h, Beckman Coulter Optima L-100 K, 70 Ti rotor, 38,000 rpm, *k* factor = 148) was added to the cultures and cells were cultured for an additional 24 h under normoxic and physioxic (3% O_2_) conditions. The use of ultracentrifuged medium for EV collection removed unwanted contaminating proteins that would otherwise have co-isolated with the desired EV populations that were collected. Cells were then imaged using an Axiovert 200M phase contrast inverted microscope complete with an AxioCam MR 1.4 MP camera and AxioVision Rel. 4.8.2 acquisition software (Carl Zeiss Canada Ltd., Toronto, CA) at an objective of 10×/0.25 Ph1 prior to collection of the expended (i.e., conditioned) medium. 3% O_2_ in the headspace was maintained using a SubChamber system (BioSpherix, Parish, NY) and displacing O_2_ by injecting nitrogen through oxygen controllers (ProOx Model 110, BioSpherix, Parish, NY). The oxygen controllers were calibrated as per manufacturer protocols using the O_2_ percentage in air (20.9%) and via the flow of 100% N_2_ (0% O_2_) in a calibration chamber. Petri dishes filled with ddH_2_O were placed within the chamber to maintain appropriate humidity.

A headspace gas composition containing 3% O_2_ was chosen as it would result in the cells within the culture medium experiencing physiological oxygen levels. The oxygen concentration in the headspace is higher than that experienced by the cells due to the difference in oxygen solubility between the air and culture medium, and due to the rate of oxygen diffusion through the medium to the cells at the bottom of the flask. Henry’s law and Fick’s law can be applied to approximate the O_2_ concentration at the liquid–gas boundary, and the O_2_ concentration at the cells, respectively. 3% O_2_ in the headspace equates to the cells seeing an actual oxygen concentration of ~ 2.2% O_2_, considering an estimated O_2_ solubility of 1.26 μM O_2_ per 1 mmHg (approximated as blood plasma at 37 °C based on a similar presence of dissolved salts [45]), a diffusivity constant of 2.69 × 10^–5^ cm^2^/s (approximated for culture medium at 37 °C [46]), and a maximal oxygen consumption rate by the cells of 113 fmol/cell/h [47] (60,000 cells/cm^2^ at confluence).

### EV isolation

Differential ultracentrifugation was used to isolate the EV fractions as previously reported, using a high recovery, low specificity protocol [48]. The medium was centrifuged at 2000 g and 4 °C for 10 min to remove pelleted cell debris and apoptotic bodies, and at 10,000 g and 4 °C for 30 min to further remove remaining apoptotic bodies and larger microvesicles. The medium was then diluted with DPBS and ultracentrifuged at 105,000 g and 4 °C for 2 h (Beckman Coulter Optima L-100 K, 70 Ti rotor, 38,000 rpm, *k* factor = 148). Given that this is a high yield, low purity method, the resulting pellets containing the EVs also contained aggregates of proteins secreted by the cells during the EV collection period. Thus, the resulting EV populations collected were not pure EVs, and instead referred to as EV fractions. The pellet containing the EV fraction was resuspended in either EBM-2/EGM-2 to be used fresh in CMEC experiments, in DPBS for transmission electron microscopy (TEM) and single particle interferometric reflectance imaging sensor (SP-IRIS) analyses, or in RIPA buffer (1× with 10 μL/mL protease inhibitors (EMD Millipore, Burlington, MA)) for biomolecular analyses. Resuspended EV fractions not analyzed immediately were frozen at − 80 °C in their respective solutions for subsequent analyses, as storage at − 80 °C has demonstrated no significant differences in EV physical characteristics compared to fresh EVs [49].

### EV characterization

EVs were characterized based on their size, concentration, morphology, and protein contents using validated methods. An ExoView R100 (Nanoview, Boston, MA) SP-IRIS device was utilized for particle counts, size and concentration using EV specific markers CD81, CD63, CD9, and syntenin-1, and negative marker GRP94 according to manufacturer’s protocols. Protein analyses were performed using a Luminex-based Human Angiogenesis & Growth Factor 17-Plex Discovery Assay (Eve Technologies, Calgary, Canada). TEM was used to confirm the morphology and size of EVs using a Hitachi H7650 120 kV microscope (Hitachi High-Tech, Tokyo, Japan) mounted with a BioSprint 16 MP CCD camera (Advanced Microscopy Techniques, Woburn, MA) and corresponding AMT acquisition software. Briefly, EVs were adsorbed to formvar-coated copper mesh grids (Electron Microscopy Sciences, Hatfield, PA) for 30 min, fixed in 2.5% glutaraldehyde for 15 min, washed 2× with dH_2_O, stained with 2.6% uranyl acetate, washed 2× again with dH_2_O, and dried at room temperature before being imaged at an accelerating voltage of 60 kV.

### CMEC culture

The immortalized human CMEC line, hCMEC/D3 (Cedarlane, Burlington, Canada) was grown in endothelial growth medium (EGM-2) (Lonza, Basel, Switzerland). A total of 2 million CMECs were inoculated per T-75 tissue culture flask, and the cells were passaged at 90% confluency (2–3 days of culture) to be used in experiments. Medium was replaced on day 2.

### CMEC proliferation assay

For the proliferation assay, CMECs were first seeded at 7500 cells/cm^2^ in 96 well plates in EGM-2 and cultured under normoxic (18.4% O_2_) conditions for 12 h. After 12 h, the EGM-2 was removed and the medium was replaced with 1 of 10 conditions: (i) a control of basal medium (EBM-2); (ii) a positive control of growth medium (EGM-2), (iii and iv) EBM-2 with the addition of EVs from normoxic MSCs concentrated at 10× and 20× the original medium, (v and vi) EBM-2 with the addition of EVs from physioxic MSCs concentrated at 10 × and 20×, (vii and viii) EGM-2 with the addition of EVs from normoxic MSCs concentrated at 10× and 20×, and (ix and x) EGM-2 with the addition of EVs from physioxic MSCs concentrated at 10× and 20×. The final volume of each well was 100 μL. After 48 h, the cells were first imaged on the Axiovert 200M phase contrast inverted microscope at 10×/0.25 Ph1 objective, then washed once with DPBS and frozen at − 80 °C. The amount of DNA in each sample was quantified using a CyQUANT Cell Proliferation Assay Kit (Thermo Fisher Scientific, Waltham, MA) as per manufacturer’s instructions. Briefly, 200 μL of CyQUANT GR/cell lysis buffer was added to each well and incubated in the dark at room temperature for 5 min. Sample fluorescence was read using a microplate reader at 485 nm excitation and 525 nm emission maxima. OD readings were converted to cell number using a standard curve of known DNA concentration, and approximating DNA per cell at the weight of the human genome, 6.41 pg [50].

### CMEC tube formation assay

Twenty-four well plates were coated with 200 μL of Geltrex LDEV-Free Reduced Growth Factor Basement Membrane Matrix (Thermo Fisher Scientific, Waltham, MA) and incubated at 37 °C for a minimum of 30 min prior to use. CMECs were inoculated at 30,000 cells/cm^2^ in EBM-2 with and without the addition of EVs from normoxic or physioxic MSC cultures at concentrations of 10 × and 20× the original medium. The final well volume for each condition was 0.5 mL. Tube formation was evaluated at 3, 6 and 9 h time points, with a minimum of 3 images taken of each well on the Axiovert 200M phase contrast inverted microscope at 10×/0.25 Ph1 objective. Each image was analyzed using ImageJ Angiogenesis Analyzer software.

### RT-qPCR

MSCs were washed with DPBS, lysed in Trizol reagent and frozen at -20 °C. CMECs were inoculated at 26,000 cells/cm^2^, grown in EGM-2 for 24 h, and then the medium was replaced with EBM-2, EGM-2, or EBM-2 with the addition of EVs 20× concentrated from the original media from each condition. After another 24 h, cells were lysed using Trizol, collected and frozen at − 20 °C. Total RNA was isolated and reverse transcribed as described previously [48]. Human-specific primers were validated as listed in Table 1 and gene expression was normalized to *18S*. Resultant data was analyzed using the ^ΔΔ^CT method.

**Table 1:** Human-specific primers used for RT-qPCR (F: forward; R: reverse)

Gene	Primer Sequence (5′-3′)	Origin
*18S*	F: TGG TCG CTC GCT CCT CTC C R: CGC CTG CTG CCT TCC TTG G	NR_003286
*ANG1*	F: CCT GAT CTT ACA CGG TGC R: GCT TTC ATA ATC GCT TCT	NM_001314051
*ANG2*	F: ACG GAC CAA AGC AAG ACC R: GGT TGT GAC AGC AGC GTC	XM_017013318
*BCL2*	F: GAT GAC TGA GTA CCT GAA CC R: AGT TCC ACA AAG GCA TCC	EU287875
*FGF2*	F: CGC GGT TGC AAC GGG AT R: GGG TTC ACG GAT GGT TGT CT	NM_27968
*CLDN5*	F: TTT CCC TAA CTT CAG CTG CC R: CCC TCT TTG AAG GTT CGG G	NM_001130861
*HIF1*	F: CCA GTT ACG TTC CTT CGA TCA GT R: TTT GAG GAC TTG CGC TTT CA	NM_001243084
*HIF2*	F: GGT GGC AGA ACT TGA AGG GTT A R: GGG CAA CAC ACA CAG GAA AT	NM_001430
*HMOX1*	F: ATG ACA CCA AGG ACC AGA GC R: GTG TAA GGA CCC ATC GGA GA	NM_002133
*IL6*	F: TCA ATA TTA GAG TCT CAA CCC CCA R: TTC TCT TTC GTT CCC GGT GG	NM_000600
*MCP1*	F: GCA ATC AAT GCC CCA GTC AC R: TCT TTG GGA CAC TTG CTG CT	S71513
*OCLN*	F: GCA AAG TGA ATG ACA AGC GG R: CAC AGG CGA AGT TAA TGG AAG	NM_002538
*SDF1*	F: GGA CTT TCC GCT AGA CCC AC R: GCC CGA TCC CAG ATC AAT GT	NM_199168
*TGFB1*	F: GGG GAA ATT GAG GGC TTT CG R: CCA GGA CCT TGC TGT ACT GC	NM_000660
*VEGF*	F: ACG GTC CCT CTT GGA ATT GG R: GGC CGC GGT GTG TCT A	M32977
*TJP1*	F: TGC TGA GTC CTT TGG TGA TG R: AAT TTG GAT CTC CGG GAA GAC	NM_003257

### Statistical methods

Data are presented as mean ± standard deviation (SD). A minimum of triplicate samples was used to evaluate conditions. Two-tailed unpaired t-tests were used to compare between two conditions, and one-way ANOVAs followed by post hoc analysis using the Bonferroni multiple comparisons test were used to compare between multiple conditions. The difference in means was determined to be significant if *p* < 0.05. GraphPad Prism was utilized to compute all statistics.

## Results

### Physioxic conditions upregulate *SDF1* and *VEGF* gene expression in MSCs

**Fig. 1:**
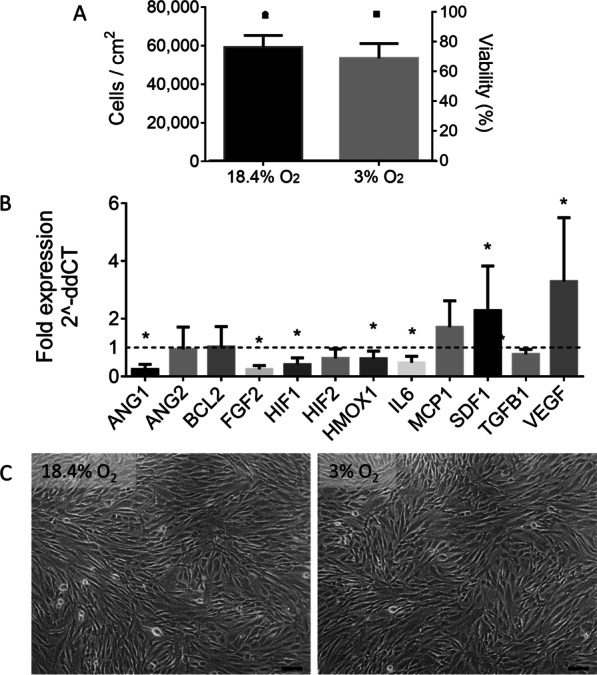
MSC growth and gene expression in normoxic (18.4% O_2_) and physioxic (3% O_2_) conditions. **A** MSC cell density and viability in normoxic and physioxic conditions after 96 h in culture. **B** Fold change in gene expression levels in MSCs cultured in physioxic as compared to normoxic environments. Expression levels are normalized to *18S*. *N* = 5, *P < 0.05, bars represent mean and SD. **C** 10× phase contrast microscopy images of MSCs after culture in normoxic and physioxic conditions for 24 h (total 96 h in culture). Scale bar = 100 μm

To evaluate the effect of physioxia on MSC characteristics, MSC growth and angiogenic gene expression were measured under normoxia and physioxia. MSCs were cultured in normoxic (18.4% O_2_) conditions for 72 h, at which point the cells were washed and exposed to EV collection medium and placed back into either normoxic or physioxic (3% O_2_) conditions for an additional 24 h. Differences in cell number and gene expression were evaluated after a total of 96 h of culture, as shown in Fig. 1. MSC numbers were not significantly different between the two conditions (59 ± 6 × 10^3^ cells/cm^2^ in normoxia, 53 ± 8 × 10^3^ cells/cm^2^ in physioxia), nor was the cell viability (98.4 ± 2.1% in normoxia, 98.6 ± 1.1% in physioxia). In physioxic cultured MSCs, expression of the gene encoding vascular endothelial growth factor (*VEGF*) was significantly upregulated (3.3 ± 2.2 fold), as was that of stromal cell-derived factor 1 (*SDF1*, 2.3 ± 1.5 fold). Expression levels of genes encoding angiopoietin-1 (*ANG1*), basic fibroblast growth factor (*FGF2*), hypoxia-inducible factor-1α (*HIF1*), heme oxygenase-1 (*HMOX1*), and interleukin-6 (*IL6*) were significantly downregulated in physioxia. There were no significant differences between conditions in the expression of genes encoding angiopoietin-2 (*ANG2*), B-cell lymphoma 2 (*BCL2*), hypoxia-inducible factor-2α (*HIF2*), monocyte chemoattractant protein-1 (*MCP1*), or transforming growth factor β-1 (*TGFB1*). No changes in cell viability or morphology were detected between the two conditions.

### Physioxia alters the angiogenic profile of MSC-derived EVs

**Fig. 2:**
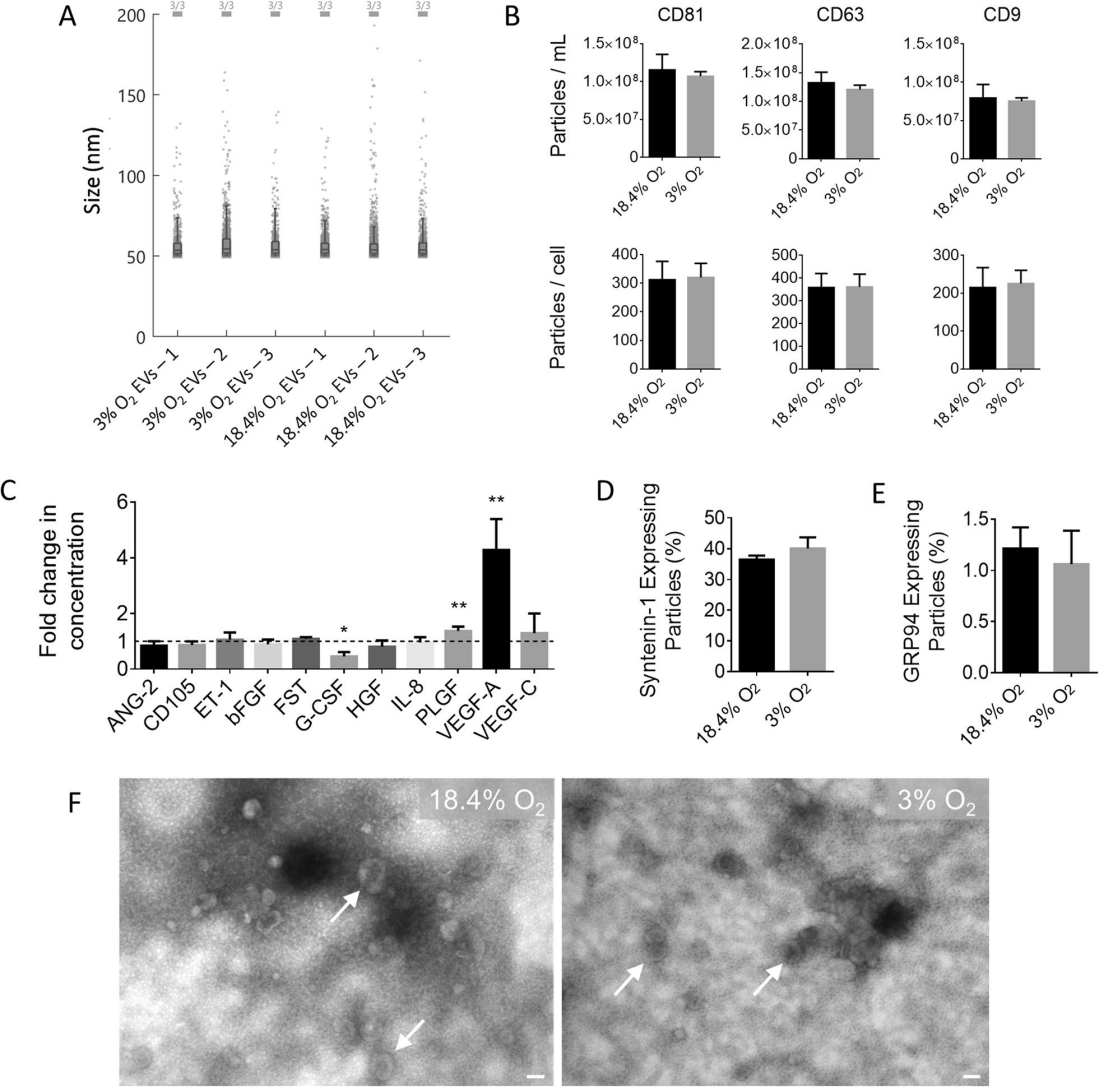
Characterization of EVs isolated from MSCs cultured under normoxic (18.4% O_2_) and physioxic (3% O_2_) conditions. **A** Size distribution measured by SP-IRIS of particles in EV fractions isolated from normoxic and physioxic MSC cultures. **B** Number of CD63, CD81, and CD9 expressing particles isolated from normoxic and physioxic MSC cultures as quantified by SP-IRIS (*N* = 3). Total concentration is expressed as particles/ml normalized to CM and EV yield is expressed as particles/cell. **C** Biomolecule concentrations for EV fractions from normoxic and physioxic cultures as measured using Luminex (*N* = 3). **D** % of syntenin-1 expressing particles as measured by % of total CD63 expressing particles (*N* = 3). **E** % of GRP94 expressing particles as measured by % of total CD63 expressing particles (*N* = 3). **F** Representative TEM images of normoxic and physioxic samples taken at 50,000× and 60 kV, with arrows indicating typical EV cup-shape morphology. Scale bar = 100 nm. *P < 0.05, **P < 0.01, bars represent mean and SD

To evaluate changes in EV composition and concentration, SP-IRIS, Luminex, and TEM analyses were carried out. The mean particle size was measured to be 56 ± 18 nm for EV fractions obtained from both normoxic and physioxic MSC cultures (Fig. 2A). Total EV concentration in the isolated EV fractions, as measured by particle concentrations of CD9, CD63 and CD81, was not significantly different between the different EV fractions (Fig. 2B). EV cargo analyses revealed that syntenin-1 was expressed in 36% of particles from normoxic conditions and 40% from physioxic conditions (Fig. 2D), while glucose-regulated protein 94 (GRP94) was detectable in < 2% of particles (Fig. 2E). The higher concentration of syntenin-1 in EV fractions from physioxic conditions indicates there may be a higher ratio of exosomes to microvesicles released in physioxic conditions, as syntenin-1 is considered a marker specific for exosomes [51]. TEM imaging (Fig. 2F) revealed that EVs displayed characteristic cup-shape morphology. Biomolecular Luminex analyses were performed on known angiogenic factors within MSC-derived EVs obtained from passage 7, day 4 cultures (Fig. 2C). Production of VEGF-A was significantly higher (4.3 ± 1.1 fold) in EV fractions from physioxic cultures compared to those from normoxic cultures, as was placental growth factor (PLGF, 1.4 ± 0.16 fold), while granulocyte colony-stimulating factor (G-CSF, 0.46 ± 0.16 fold) was reduced. No significant differences were found in concentrations of ANG-2, basic fibroblast growth factor (bFGF), endoglin (CD105), endothelin-1 (ET-1), follistatin (FST), hepatocyte growth factor (HGF), interleukin-8 (IL-8), or VEGF-C. The absolute concentrations of angiogenic factors found in the EV fractions can be found in Additional file 1: Table S1.

### MSC-derived EVs enhance angiogenic functionality of CMECs

**Fig. 3:**
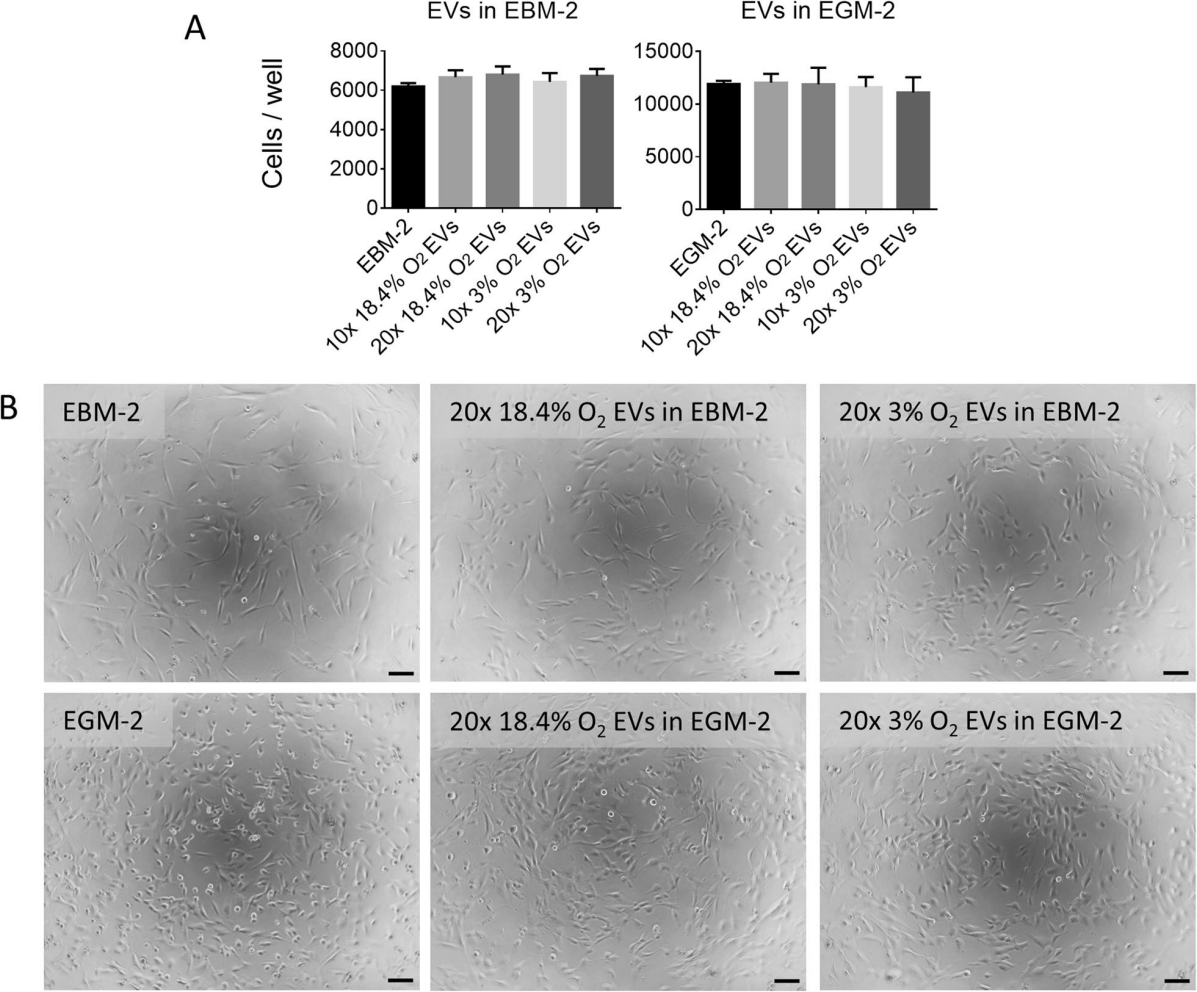
Effect of EVs isolated from MSCs cultured in normoxic (18.4% O_2_) and physioxic (3% O_2_) conditions on CMEC proliferation. **A** Number of cells per well as measured by a CyQUANT Proliferation assay for CMECs under normoxic and physioxic conditions after 48 h. *N* = 3, *P < 0.05, ***P < 0.001, bars represent mean and SD. **B** 10× phase contrast images of CMECs after 48 h of culture with treatments in EBM-2 (negative control), EGM-2 (positive control), and EBM-2 or EGM-2 supplemented with EVs at 20× original medium concentrations from normoxic and physioxic MSC cultures. Scale bar = 100 μm

EV fractions isolated from MSC cultures were assessed for their efficacy in inducing proliferation and tube formation of CMECs. To measure EV efficacy in inducing CMEC proliferation, isolated EVs from normoxic and physioxic MSC cultures were resuspended in either endothelial basal medium (EBM-2) or endothelial growth medium (EGM-2). EGM-2 consisted of EBM-2 with the addition of growth supplements including fetal bovine serum (FBS) and proliferative factors such as bFGF. EGM-2 was included in this study as it is the standard medium used to culture CMECs. However, an important consideration is that the known stimulatory impacts of FBS and proliferative factors in EGM-2 could obscure the full impact of the supplemented EVs. Thus, EBM-2 medium was also included in this study to better observe the impact of the supplemented EVs on CMECs in the absence of FBS and other factors. The EV fractions were added at concentrations of 10× and 20× that of the original medium (i.e., for 10×, EVs isolated from 10 mL of CM was added per 1 mL of CMEC culture medium). 10× and 20× concentrations were chosen to evaluate dose dependence as they were found to stimulate CMEC tube formation in preliminary studies, with a maximum concentration of 20× to enable full resuspension of the EV pellet. A volume-based dosage was used due to uncertainties in currently available methods to quantify EVs. EVs resuspended in either EBM-2 or EGM-2 had no observable effect on CMEC proliferation (Fig. 3). Images for 10× EV conditions can be found in Additional file 2: Fig. S1.

**Fig. 4:**
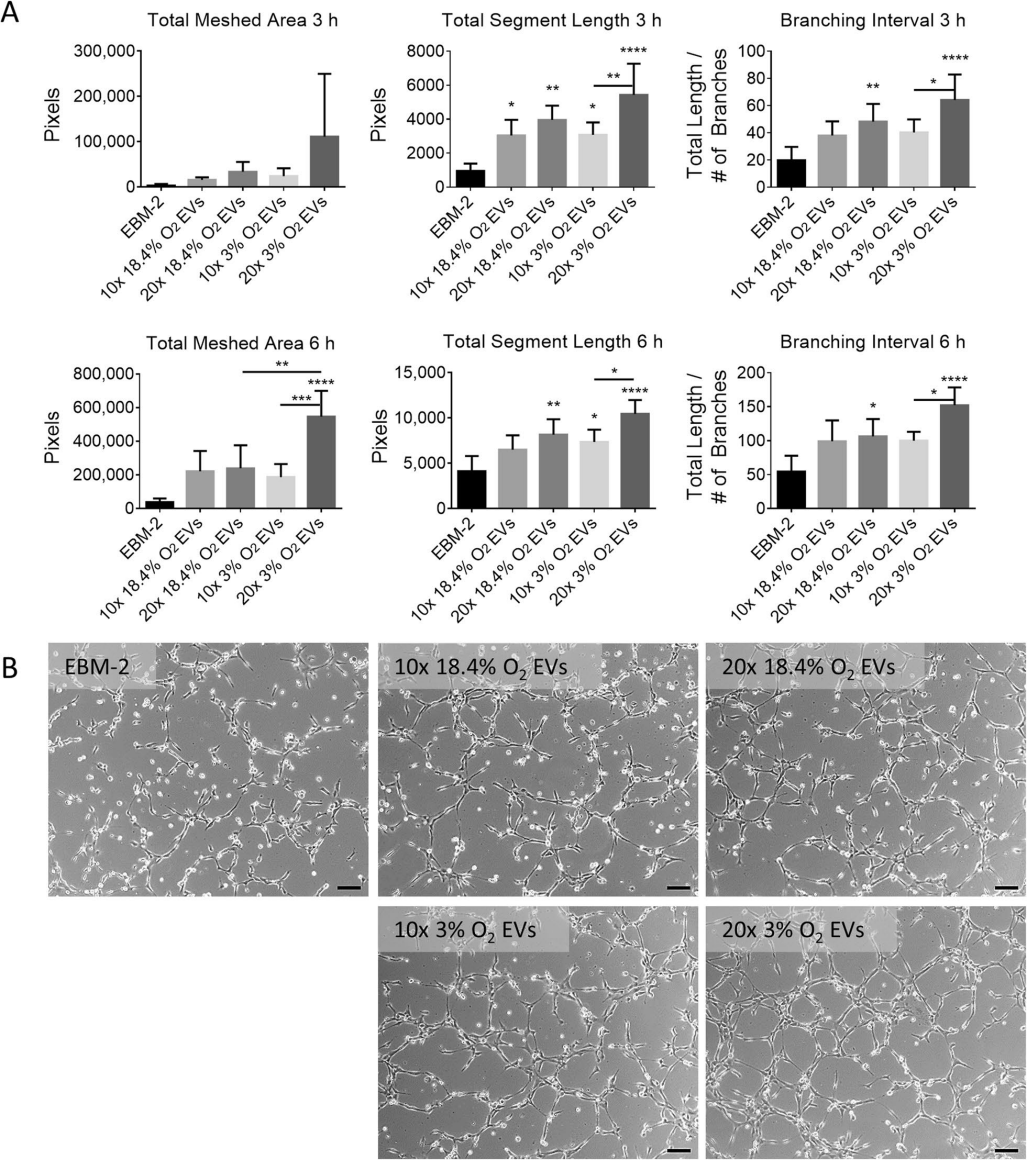
Effect of EVs isolated from MSCs cultured in normoxic (18.4% O_2_) and physioxic (3% O_2_) conditions on CMEC tube formation. **A** Total meshed area, total segment length and branching interval as measured by ImageJ for CMECs cultured on Geltrex and exposed to EBM-2 (negative control), or EBM-2 supplemented with EVs at 10× and 20× original medium concentrations from normoxic or physioxic MSC cultures. Total meshed area represents the total area fully enclosed by tubes. Total segment length represents the sum of the total length of tube segments analyzed. Branching interval represents the mean distance that separates two branches in the analyzed area. *N* = 3–7, *P < 0.05, **P < 0.01, ***P < 0.001, ****P < 0.0001, bars represent mean and SD. **B** 10× phase contrast images of CMECs on Geltrex at 6 h in each of the conditions. Scale bar = 100 μm

EVs were further evaluated for their stimulatory effects on CMEC tube formation. Tube formation was evaluated at 3, 6, and 9 h post-inoculation and quantified using the ImageJ Angiogenesis Analyzer. Tube formation was measured by total meshed area, total segment length and branching interval (segment length per branch), as shown in Fig. 4-A (see Additional file 3: Fig. S2 for 9 h data). At 3 h, all EV conditions exhibited significantly higher tube formation compared to the control EBM-2 in a dose dependent manner. At 6 h, the presence of physioxic EVs resulted in a significantly higher meshed area and branching interval compared to normoxic EVs, demonstrating that physioxic EVs have a higher stimulatory effect on tube formation in vitro, which suggests that physioxic EVs may better promote angiogenesis compared to normoxic EVs. Similarly, the number of isolated segments was reduced for all time points with EV treatment (see Additional file 4: Figure S3), further indicating that EVs contribute to more organized tube formation in CMECs. This effect is prominent in images taken at 6 h (Fig. 4B).

### MSC-derived EVs upregulate the expression of angiogenic genes in CMECs

**Fig. 5:**
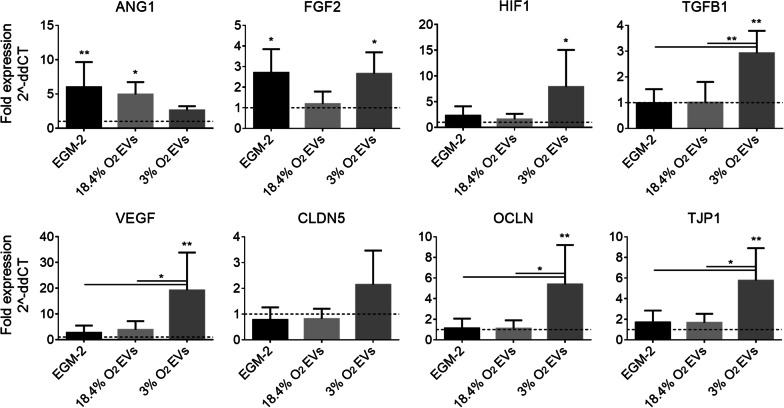
Effect of EVs isolated from MSCs cultured in normoxic (18.4% O_2_) and physioxic (3% O_2_) conditions on CMEC angiogenic and tight junction gene expression. Fold change in gene expression of *ANG1*, *FGF2*, *HIF1*, *TGFB1*, *VEGF*, claudin-5 (*CLDN5*), *OCLN*, and *TJP1* normalized to *18S* for CMECs exposed to EGM-2 or 20× EV conditions compared to control EBM-2. Statistical significance shown in comparison to control. *N* = 5, *P < 0.05, **P < 0.01, bars represent mean and SD

Changes to CMEC expression of genes specific to angiogenesis and BBB function resulting from culture with MSC-EVs were evaluated. CMECs were cultured with the addition of MSC-derived EVs from normoxic and physioxic cultures suspended in EBM-2 medium at a concentration of 20× the original medium, as 20× was found to induce significant effects in prior experiments. Cells in EGM-2 were analyzed as a positive control to ensure differences in expression were due to EV supplementation and not a result of a loss of cell function in non-supplemented basal medium. The cells were washed and lysed with Trizol after 24 h and frozen at − 80 °C for subsequent RT-qPCR analyses. The expression of angiogenic genes *FGF2*, *HIF1*, *TGFB1* and *VEGF* was upregulated in CMECs treated with physioxic EVs, in addition to genes encoding for proteins related to the maintenance of the BBB, occludin (*OCLN*) and tight junction protein-1 (*TJP1*), compared to EBM-2 controls (Fig. 5). EVs from normoxic MSC cultures resulted in a significant increase in *ANG1* only.

## Discussion

Since the benefits of MSC-EVs in a rat model of stroke were first reported in 2013 [52], several studies have suggested that MSC-EVs have therapeutic potential for angiogenesis, neurogenesis, neurological recovery, neuroprotection, immunomodulation, reduction of infarct volume, neuroplasticity and white matter recovery [15]. Physiologically, it has been reported that stroke triggers the mobilization of CD105^+^ microparticles (regarded as MSC-EVs) and ischemia induces an increase in both circulating and regional levels of EVs [53], thereby leading to the hypothesized therapeutic relevance of MSC-EVs in stroke, and in particular, MSC-EVs obtained from low oxygen environments. In the present studies, it was found that culturing MSCs in physioxic (3% O_2_) conditions as opposed to normoxic (18.4% O_2_) conditions resulted in the generation of MSCs with upregulated angiogenic gene expression, and subsequently, release of EV fractions containing higher concentrations of angiogenic proteins. Using an in vitro model for angiogenesis within the brain, it was determined that adipose MSC-derived EVs enhanced the tube formation activities of human CMECs, with those derived from MSCs cultured under physioxic conditions eliciting the highest response. Only EVs derived from MSCs cultured under physioxic conditions were able to induce significant changes in CMEC gene expression, increasing both angiogenic and tight junction protein expression, while EVs from normoxic cultures did not appear to significantly alter basal expression levels for these genes.

It has been previously reported that the administration of recombinant VEGF in an ischemic stroke model, and the overexpression of *HIF1* in CMECs both increased angiogenesis but resulted in the disruption of tight junction proteins leading to BBB leakage and risk of hemorrhage [54, 55]. In this study, overexpression of angiogenic factors and induced angiogenesis by MSC-EVs did not appear to disrupt tight junction proteins, further demonstrating the utility and complexity of MSC-EVs as opposed to therapeutics focused on an individual target. While other studies have evaluated MSC-EVs from low oxygen cultures in CMEC angiogenesis [43, 56], with supportive results regarding enhanced angiogenic function, they did not evaluate their effect on tight junction proteins, a key consideration for BBB functionality. Further, underlying differences in the cell source, clinical applicability of the medium used, methods for EV isolation, and methods of analysis represent the significance of the current study.

The current study utilized MSCs derived from adipose tissue due to their ease of isolation and the availability of the tissue [57], as well as their enhanced angiogenic potential as compared to other easily sourced tissues such as bone marrow and Wharton-jelly [7]. A clinically applicable chemically defined serum-free culture medium, PPRF-msc6 was used throughout the process, which was previously developed specifically for the culture of MSCs [35]. The removal of serum from culture media is an important consideration, as it can contain adventitious infectious agents, and the batch-to-batch variability can undermine the reproducibility of cell production processes [58]. This also applies to the generation of cell-derived EV populations for clinical translation, with an added consideration being contamination with the EVs naturally present in serum. Thus, stringent culture procedures utilizing defined serum-free media are essential to promote process reproducibility and prevent the co-isolation of serum-derived EVs. Another consideration is that many serum-free media used for cell culture still contain serum-derived proteins, such as albumin and fetuin, which commonly co-isolate with the desired EV populations, thereby potentially influencing functionality testing of those EVs [59]. To minimize this possibility, and in alignment with the recommendations by ISEV [60], the EV collection medium used in the current study was ultracentrifuged to eliminate any contaminating proteins.

With the utilization of ultracentrifuged EV collection medium, induction of CMEC proliferation by MSC-EVs was not detected. In supplemental experiments, it was demonstrated that PPRF-msc6 medium, conditioned medium (CM), and the supernatant (SN) obtained after isolating EV pellets enhanced CMEC proliferation (see Additional file 5: Fig. S4). EVs isolated from PPRF-msc6 medium that had not been ultracentrifuged prior to use, similarly improved CMEC proliferation, demonstrating that factors within PPRF-msc6 media do indeed influence functional results. Alternatively, in the same experiments, it was found that SN had no significant effect but appeared to reduce tube formation of CMECs, while the EV fractions isolated under non-ultracentrifuged medium showed similar improvements in tube formation as seen in this study (see Additional file 6: Fig. S5).

A large body of research has focused on the RNAs present in EVs, with several studies demonstrating uptake and subsequent gene regulation by EV transported miRNAs [61, 62]. Further work has demonstrated the upregulation of specific angiogenesis-promoting miRNAs in response to hypoxia [43, 56]. However, some studies have challenged this idea, hypothesizing that the low concentration of miRNAs, their underlying structure, and the absence of accessory proteins (i.e., argonaute proteins) necessary for their functionality within MSC-EVs, may discredit their therapeutic benefits being related to their contained RNAs [63–65]. The observed inconsistencies between such studies are likely due to the limitations of current methodologies applied for EV isolation and miRNA detection. Indeed, a key limitation in current EV studies, including this one, is the lack of existing and standardized isolation methods that can isolate a pure population of EVs with high efficiency [60]. Isolated EV pellets contain aggregates of proteins, nucleic acids, and lipids secreted by the cells during the EV collection period, which co-isolate with EVs due to similarities in size/density, and/or due to molecular interactions at the EV surface [60]. It is largely unknown whether these co-isolated molecules contribute to, or even improve EV uptake and/or functionality, as evidenced by the reduced uptake and function of EVs following surface protein and DNA digestion [66–68]. A thorough understanding of EV cargo and how it changes with different culture conditions, and the development of new technologies to better characterize these populations, will be of benefit to the field, and is ultimately necessary in fully characterizing EV populations.

We focused on angiogenic gene expression of parent MSCs and the corresponding angiogenic protein content in the EV fraction to predict therapeutic efficacy. The genes and proteins chosen are well established in the literature for promoting angiogenesis and serve as a strong basis of comparison between normoxic and physioxic conditions. Our findings were consistent with that of the current literature, in which Anderson et al*.* [69] found that angiogenic pathways were upregulated in bone marrow MSCs exposed to hypoxic conditions and similarly, Zhang et al*.* [10] reported higher protein content in microvesicles derived from adipose MSCs grown in hypoxia. The importance of culturing MSCs in physiological oxygen concentrations is well reported [18, 70]. Reduced oxygen concentrations in culture have been shown to stabilize HIF-1α, thereby mediating the expression of several genes that promote angiogenesis, prevent apoptosis, and induce migration and homing of these cells to sites of ischemia. In this study, the downregulation of *HIF1* was unexpected and could be due to HIF-dependent induction of prolyl hydroxylase domain containing proteins (PHD2 and PHD3) downregulating HIF activity in prolonged (overnight) hypoxia, as reported by Marxsen et al. [71].

Downstream targets of *HIF1* include *VEGF*, *SDF1*, *FGF2*, and *HMOX1* [72–75]. In physioxic cultures, enhanced expression and secretion of *VEGF* was detected, the key mediator of angiogenesis and neuroprotective agent in stroke [76], and enhanced expression of *SDF1*, an important regulator of angiogenesis that attracts cells expressing *CXCR4* such as neuroblasts and endogenous MSCs to migrate to the area [77], consistent with the literature [22, 30, 72, 78–81]. On the contrary, a reduction in *FGF2* and *HMOX1* expression was observed in physioxic MSCs in the present study. bFGF protein content in the EV fractions were similar, indicating more protein per mRNA was produced in physioxic conditions, consistent with a study by Conte et al*.* [82], which found a significant reduction of *FGF2* mRNA levels under short-term ischemic conditions, but a corresponding increase of bFGF at the translational level. The effect of low oxygen conditions, or physioxia, at both the transcriptional and translational level of many of these factors differs throughout the literature and may be due to donor heterogeneities, differing growth media or time of harvest, or time of exposure [17]. There is a need to further explore donor heterogeneity under controlled culture conditions to determine whether such responses are repeatable among donors. Despite such heterogeneities at the transcriptional and translational level, the reported functional effects of EVs are more consistent, and therefore merit the use of functional models to test for EV efficacy.

Due to the inherent limitations of EV isolation and detection methodologies at this time, a specific mechanism of action of MSC-EVs was not evaluated in this study. Instead, the impact of the collected EVs was evaluated in aggregate, termed the EV fraction, and considered to act as a whole bioactive drug. With the future development of more defined and robust isolation protocols, the specific mechanism of action will be able to be better understood. While previous in vivo studies have provided sufficient evidence of the overall neurological benefit of EVs, we were able to show that MSC-EVs could specifically induce a positive effect on CMECs, and that EVs derived from MSCs cultured in physioxic conditions are functionally of higher relevance. There is still a significant need to evaluate optimal dosing and time of treatment in clinical models, better understand limitations in donor heterogeneity, and to develop a scalable solution for EV production specific to the application of cerebral ischemia. The in vitro angiogenesis CMEC model tested offers preliminary proof-of-concept results that suggest benefits of physioxic MSC-EVs in applications of cerebral ischemia. Despite the widespread use of this model, it was limited in that it does not provide a whole system approach and may not accurately reflect in vivo conditions. As such, further studies should aim to test physioxic EVs in preclinical models of cerebral ischemia (the most relevant being middle cerebral artery occlusion) that can better provide a full system understanding. Further, more robust models should be used to further examine their effect specifically on BBB functionality and confirm the results of the gene expression analyses done in this study. For example, a Transwell apparatus may be used to assess barrier permeability based on the transport of tracer substances, or by measurement of trans-endothelial electrical resistance [83]. Overall, the present study illustrates that well-defined culture conditions can lead to further understanding of EV production and functionality, and further the clinical relevance of MSC-EVs.

## Conclusions

This study demonstrated that a physiological oxygen environment enhances the gene expression of *VEGF* and *SDF1* in MSCs and the subsequent secretion of VEGF within the EV fraction. Furthermore, it was demonstrated that MSC-derived EVs induce angiogenic changes in CMECs without damaging their BBB functionality, in particular those isolated from physioxic cultures, and thus may have clinical utility in repair and maintenance following cerebral ischemia.

### Supplementary Information

**Additional file 1**. **Table S1 **Concentrations of angiogenic proteins in EV fractions from normoxic (18.4% O_2_) and physioxic (3% O_2_) MSC cultures.

**Additional file 2**. **Fig. S1** Phase contrast images of CMECs after 48 h of culture with treatments in EBM-2 or EGM-2 supplemented with EVs at 10× original medium concentrations from normoxic (18.4% O_2_) and physioxic (3% O_2_) MSC cultures. Scale bar = 100 μm.

**Additional file 3**. **Fig. S2** Total meshed area, total segment length and branching interval as measured by ImageJ for CMECs exposed to EVs isolated from MSCs cultured in normoxic (18.4% O_2_) or physioxic (3% O_2_) conditions. *N *= 3-7, *P < 0.05, **P < 0.01, ***P < 0.001, ****P < 0.0001, bars represent mean and SD.

**Additional file 4**. **Fig. S3** Isolated segment length measured at 3, 6 and 9 h by ImageJ for CMECs exposed to EVs isolated from MSCs cultured in normoxic (18.4% O_2_) or physioxic (3% O_2_) conditions. *N *= 3-7, *P < 0.05, **P < 0.01, ***P < 0.001, ****P < 0.0001, bars represent mean and SD.

**Additional file 5**. **Fig. S4** Effect of EVs isolated from MSCs cultured in PPRF-msc6 under normoxic (18.4% O2) or physioxic (3% O_2_) conditions on CMEC proliferation. A) OD readings from CyQUANT Proliferation assay for CMECs in EBM-2 (negative control), EGM-2 (positive control), EBM-2 supplemented with EVs at 5×, 10× or 20× original medium concentrations from normoxic and physioxic MSC cultures, EBM-2 supplemented with CM at 10×, and EBM-2 supplemented with EV-free CM (supernatant, SN) at 10×. *N* = 3, *P < 0.05, **P < 0.01, ***P < 0.001, bars represent mean and SD. B) 10× microscope images of CMECs on day 2 (48 h) under normoxia in EBM-2 (negative control), EGM-2 (positive control), and EBM-2 supplemented with EVs at 5× and 10× original medium concentrations from normoxic and physioxic MSC cultures. Scale bar = 200 μm.

**Additional file 6.** **Fig. S5** Effect of EVs isolated from MSCs cultured in PPRF-mcs6 under normoxic (18.4% O_2_) or physioxic (3% O_2_) conditions on CMEC tube formation. A) Total segment length as measured by ImageJ for CMECs at 4 and 12 h. *N* = 3-7, *P < 0.05, **P < 0.01, bars represent mean and SD. B) Branching interval (total length per branch) as measured by ImageJ for CMECs at 4 and 12 h C) 10x microscope images of CMECs on Matrigel at 12 h under normoxia in EBM-2 (control), in EBM-2 supplemented with EVs at 10× and 20× original medium concentrations from normoxic and physioxic MSC cultures, or in EBM-2 supplemented with EV-free conditioned medium (supernatant, SN) at 10x concentration. Scale bar = 200 μm.

## Data Availability

All data generated or analyzed during this study are included in this published article [and its supplementary information files].

## Abbreviations

ANG: Angiopoietin
BBB: Blood–brain barrier
BCL: B-cell lymphoma
bFGF: Basic fibroblast growth factor
CD105: Endoglin
CLDN: Claudin
CMEC: Cerebral microvascular endothelial cell
DPBS: Dulbecco’s phosphate-buffered solution
EBM: Endothelial basal medium
EGM: Endothelial growth medium
ET: Endothelin
EV: Extracellular vesicle
FBS: Fetal bovine serum
FGF: Fibroblast growth factor
FST: Follistatin
G-CSF: Granulocyte colony-stimulating factor
GRP: Glucose-regulated protein
HGF: Hepatocyte growth factor
HIF: Hypoxia-inducible factor
HMOX: Heme oxygenase
IL: Interleukin
ISEV: International Society for Extracellular Vesicles
MCP: Monocyte chemoattractant protein
miRNA: MicroRNA
MSC: Mesenchymal stem cell
OCLN: Occludin
PLGF: Placental growth factor
SD: Standard deviation
SDF: Stromal-derived factor
SP-IRIS: Single particle interferometric reflectance imaging sensor
TEM: Transmission electron microscopy
TGF: Transforming growth factor
TGP1: Tight junction protein-1
VEGF: Vascular endothelial growth factor
