# Supplementary material for: Physiological oxygen conditions enhance the angiogenic properties of extracellular vesicles from human mesenchymal stem cells
Source: Stem Cell Res Ther. 2023 Aug 23;14:218. doi: 10.1186/s13287-023-03439-9 (PMC10463845; doi:10.1186/s13287-023-03439-9)
Supplement: Supplementary file 1 — Additional file 1. Table S1 Concentrations of angiogenic proteins in EV fractions from normoxic (18.4% O2) and physioxic (3% O2) MSC cultures. [file 13287_2023_3439_MOESM1_ESM.docx]

Table S1: Concentrations of angiogenic proteins in EV fractions from normoxic (18.4% O_2_) and physioxic (3% O_2_) MSC cultures.

| **Growth Factor** | **18.4% O_2_** | **3% O_2_** |
| --- | --- | --- |
| ANG-2 | 11.44 ± 2.01 | 9.62 ± 0.76 |
| CD105 | 2296.75 ± 286.03 | 1994.88 ± 168.52 |
| ET-1 | 1.45 ± 0.35 | 1.54 ± 0.05 |
| bFGF | 1053.02 ± 182.28 | 937.27 ± 83.68 |
| Follistatin | 323.04 ± 6.67 | 351.97 ± 18.24 |
| G-CSF | 50.37 ± 11.18 | 23.06 ± 5.93 |
| HGF | 3268.76 ± 433.83 | 2631.40 ± 657.40 |
| IL-8 | 885.54 ± 124.32 | 849.63 ± 118.18 |
| PLGF | 2.22 ± 0.25 | 3.05 ± 0.08 |
| VEGF-A | 1132.97 ± 236.98 | 4849.10 ± 236.98 |
| VEGF-C | 19.05 ± 6.12 | 24.63 ± 10.91 |

Note: Presented as mean ± standard deviation. Samples are at 40x concentration.
