# Supplementary figures and images for: Physiological oxygen conditions enhance the angiogenic properties of extracellular vesicles from human mesenchymal stem cells
Source: Stem Cell Res Ther. 2023 Aug 23;14:218. doi: 10.1186/s13287-023-03439-9 (PMC10463845; doi:10.1186/s13287-023-03439-9)

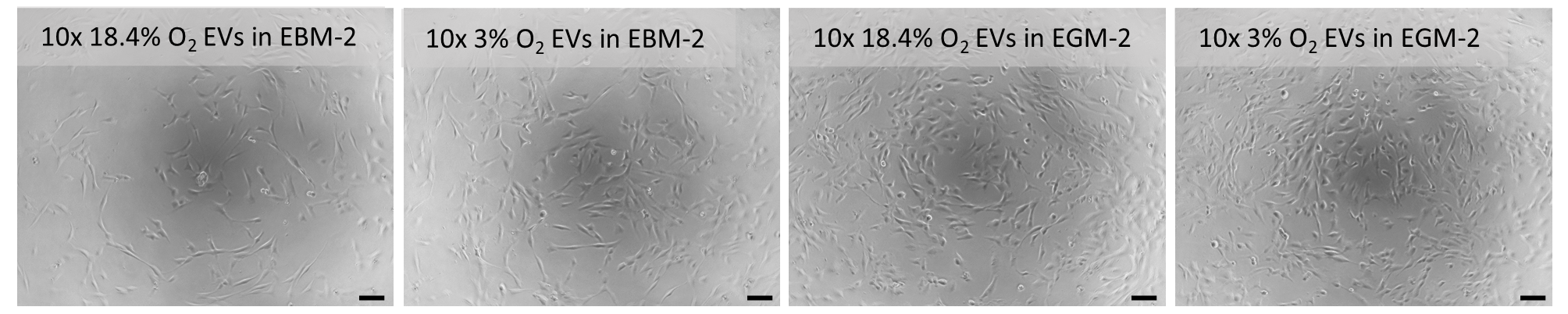

Supplement: Supplementary file 2 — Additional file 2. Fig. S1 Phase contrast images of CMECs after 48 h of culture with treatments in EBM-2 or EGM-2 supplemented with EVs at 10× original medium concentrations from normoxic (18.4% O2) and physioxic (3% O2) MSC cultures. Scale bar = 100 μm. [file 13287_2023_3439_MOESM2_ESM.tif]

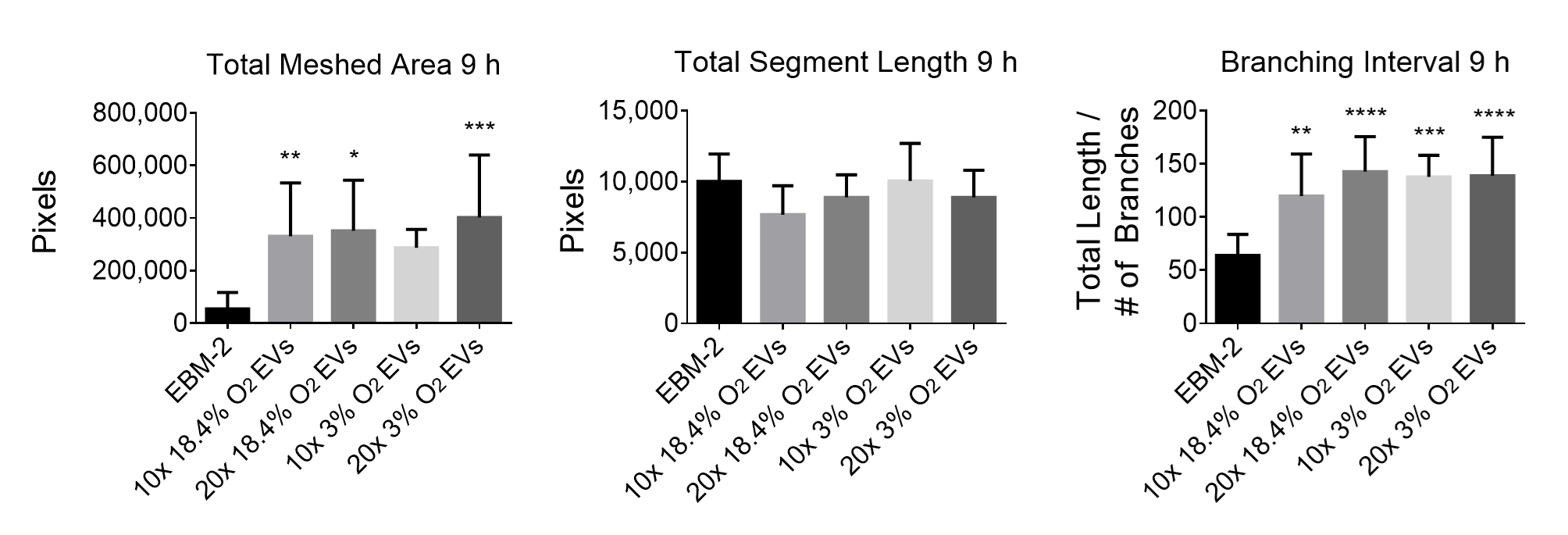

Supplement: Supplementary file 3 — Additional file 3. Fig. S2 Total meshed area, total segment length and branching interval as measured by ImageJ for CMECs exposed to EVs isolated from MSCs cultured in normoxic (18.4% O2) or physioxic (3% O2) conditions. N = 3-7, *P < 0.05, **P < 0.01, ***P < 0.001, ****P < 0.0001, bars represent mean and SD. [file 13287_2023_3439_MOESM3_ESM.tif]

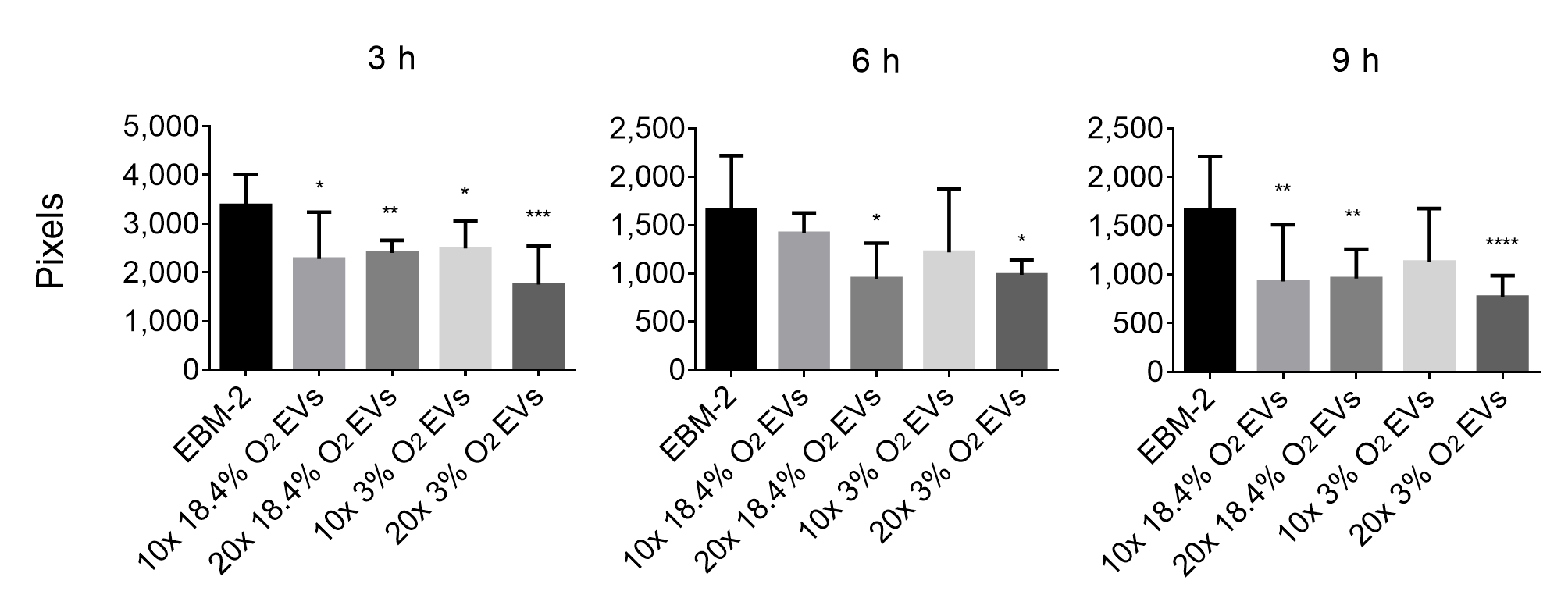

Supplement: Supplementary file 4 — Additional file 4. Fig. S3 Isolated segment length measured at 3, 6 and 9 h by ImageJ for CMECs exposed to EVs isolated from MSCs cultured in normoxic (18.4% O2) or physioxic (3% O2) conditions. N = 3-7, *P < 0.05, **P < 0.01, ***P < 0.001, ****P < 0.0001, bars represent mean and SD. [file 13287_2023_3439_MOESM4_ESM.tif]

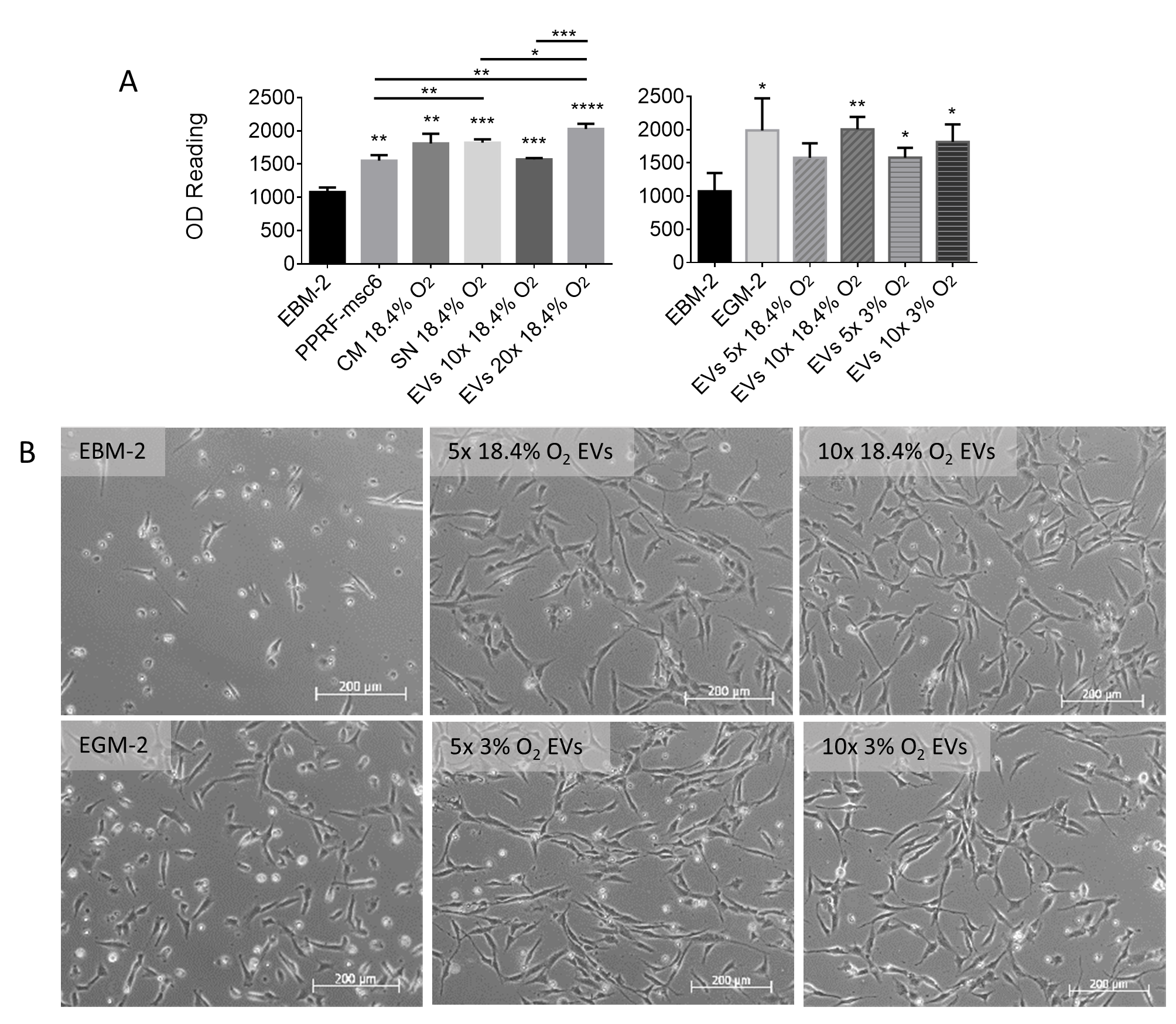

Supplement: Supplementary file 5 — Additional file 5. Fig. S4 Effect of EVs isolated from MSCs cultured in PPRF-msc6 under normoxic (18.4% O2) or physioxic (3% O2) conditions on CMEC proliferation. A) OD readings from CyQUANT Proliferation assay for CMECs in EBM-2 (negative control), EGM-2 (positive control), EBM-2 supplemented with EVs at 5×, 10× or 20× original medium concentrations from normoxic and physioxic MSC cultures, EBM-2 supplemented with CM at 10×, and EBM-2 supplemented with EV-free CM (supernatant, SN) at 10×. N = 3, *P < 0.05, **P < 0.01, ***P < 0.001, bars represent mean and SD. B) 10× microscope images of CMECs on day 2 (48 h) under normoxia in EBM-2 (negative control), EGM-2 (positive control), and EBM-2 supplemented with EVs at 5× and 10× original medium concentrations from normoxic and physioxic MSC cultures. Scale bar = 200 μm. [file 13287_2023_3439_MOESM5_ESM.tif]

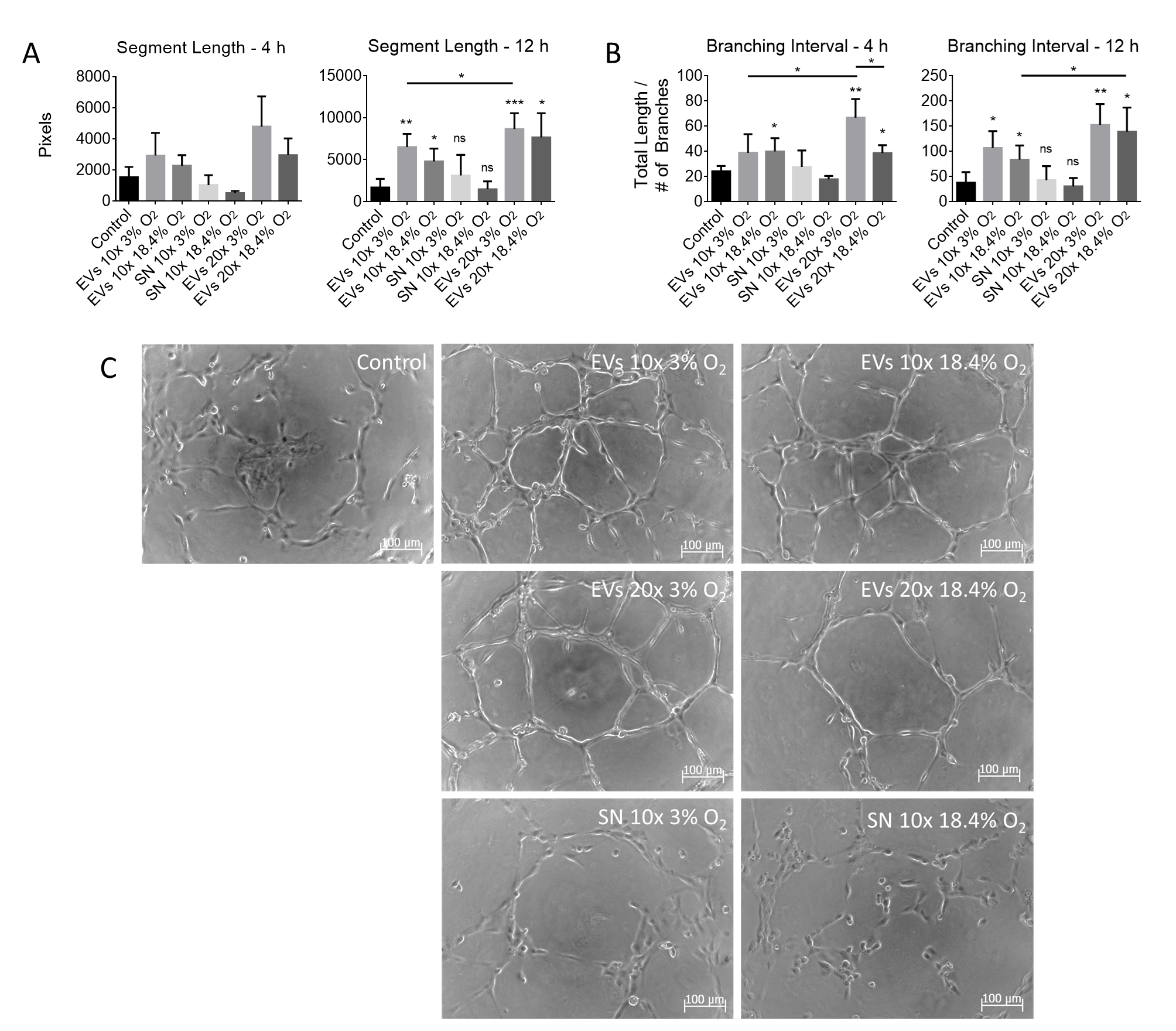

Supplement: Supplementary file 6 — Additional file 6. Fig. S5 Effect of EVs isolated from MSCs cultured in PPRF-mcs6 under normoxic (18.4% O2) or physioxic (3% O2) conditions on CMEC tube formation. A) Total segment length as measured by ImageJ for CMECs at 4 and 12 h. N = 3-7, *P < 0.05, **P < 0.01, bars represent mean and SD. B) Branching interval (total length per branch) as measured by ImageJ for CMECs at 4 and 12 h C) 10x microscope images of CMECs on Matrigel at 12 h under normoxia in EBM-2 (control), in EBM-2 supplemented with EVs at 10× and 20× original medium concentrations from normoxic and physioxic MSC cultures, or in EBM-2 supplemented with EV-free conditioned medium (supernatant, SN) at 10x concentration. Scale bar = 200 μm. [file 13287_2023_3439_MOESM6_ESM.tif]
